# Combined small RNA and degradome sequencing reveals complex microRNA regulation of catechin biosynthesis in tea (*Camellia sinensis*)

**DOI:** 10.1371/journal.pone.0171173

**Published:** 2017-02-22

**Authors:** Ping Sun, Chunzhen Cheng, Yuling Lin, Qiufang Zhu, Jinke Lin, Zhongxiong Lai

**Affiliations:** 1 College of Horticulture, Fujian Agriculture and Forestry University, Fuzhou, China; 2 Anxi College of Tea Science, Fujian Agriculture and Forestry University, Fuzhou, China; Nanjing Agricultural University, CHINA

## Abstract

MicroRNAs are endogenous non-coding small RNAs playing crucial regulatory roles in plants. Tea, a globally popular non-alcoholic drink, is rich in health-enhancing catechins. In this study, 69 conserved and 47 novel miRNAs targeting 644 genes were identified by high-throughout sequencing. Predicted target genes of miRNAs were mainly involved in plant growth, signal transduction, morphogenesis and defense. To further identify targets of tea miRNAs, degradome sequencing and RNA ligase-mediated rapid amplification of 5’cDNA ends (RLM-RACE) were applied. Using degradome sequencing, 26 genes mainly involved in transcription factor, resistance protein and signal transduction protein synthesis were identified as potential miRNA targets, with 5 genes subsequently verified. Quantitative real-time PCR (qRT-PCR) revealed that the expression patterns of novel-miR1, novel-miR2, csn-miR160a, csn-miR162a, csn-miR394 and csn-miR396a were negatively correlated with catechin content. The expression of six miRNAs (csn-miRNA167a, csn-miR2593e, csn-miR4380a, csn-miR3444b, csn-miR5251 and csn-miR7777-5p.1) and their target genes involved in catechin biosynthesis were also analyzed by qRT-PCR. Negative and positive correlations were found between these miRNAs and catechin contents, while positive correlations were found between their target genes and catechin content. This result suggests that these miRNAs may negatively regulate catechin biosynthesis by down-regulating their biosynthesis-related target genes. Taken together, our results indicate that miRNAs are crucial regulators in tea, with the results of 5’-RLM-RACE and expression analyses revealing the important role of miRNAs in catechin anabolism. Our findings should facilitate future research to elucidate the function of miRNAs in catechin biosynthesis.

## 1. Introduction

MicroRNAs, a class of small non-protein-coding RNAs, play essential roles in post-transcriptional regulation and many other biological processes in animals and plants [1, 2]. Based on the base-pair complementary mechanism, miRNAs recognize and combine the target genes to regulate their expression mainly in two ways: direct cleavage of target mRNAs and inhibition of target gene transcription [3]. Studies have shown that miRNAs are involved in a variety of plant growth and development processes, such as root, leaf and flower morphogenesis [4], signal transduction, hormone responses [5, 6] and nutrient metabolism [7]. Plant miRNAs are mainly identified by cloning, bioinformatic prediction, high-throughput sequencing and Northern blotting. The most popular of these techniques is high-throughput sequencing because of its affordability and easier and faster access to large numbers of miRNAs, especially those in low abundance [8]. Because miRNAs in plants usually act by cleaving their target genes or inhibiting their expression, target gene determination is crucial for miRNA functional analysis. Although miRNA target genes can be predicted using bioinformatic methods, they are mainly identified by degradome sequencing or by RNA ligase-mediated rapid-amplification of 5’cDNA ends (5’-RLM-RACE). These two methods can also be used to identify target genes of miRNA cleavage sites, which increase their utility for miRNA functional analysis.

Tea (*Camellia sinensis*), rich in catechins, is one of the world’s most popular non-alcoholic beverages. Catechins, an important group of compounds in tea leaves, comprise ester-type catechins (e.g., epicatechingallate [ECG] and epigallocatechingallate [EGCG]) and non-ester-type catechins (e.g., epicatechin [EC] and epigallocatechin [EGC]). EGCG is the most abundant catechin in tea and is highly effective against cancer [9]. EGCG can restrict cancer cell growth, suppress androgen receptor functions and regulate cancer cell survival, angiogenesis and movement [10]. Based on their molecular constituents, catechins can be classified as simple catechins and ester catechins [11]. Among the many enzymes known to be involved in catechin synthesis are chalcone synthase (CHS), chalconeisomerase (CHI), dihydroflavonol 4-reductase (DFR), anthocyanidin synthase (ANS), anthocyanidinreductase (ANR) and flavonoid 3,5-hydroxylase (F3’5’H) [11–13]. Environmental factors affecting catechin accumulation include soil humidity, light intensity and nutrition [14]. Short-term ultraviolet-B irradiation promotes the accumulation of total catechins, whereas excessive irradiation suppresses it [15]. MiRNAs have been identified as important regulators of gene expression at transcriptional and post-transcriptional levels and cleavage of target mRNAs have been demonstrated to being the main regulatory method of gene expression in plants [16]. Although many conserved and novel miRNAs have been discovered by high-throughput [17] and microarray chip [18] in tea plant, the direct regulatory roles of miRNAs in catechin biosynthesis is not well defined. In this study, we therefore used high-throughput sequencing to identify conserved and novel miRNAs in tea and analyzed potential miRNA targets by 5’-RLM-RACE. To further screen for possible miRNAs involved in catechin accumulation, we conducted an expression analysis. The combination of 5’-RLM-RACE and expression analyses allowed us to discover new catechin biosynthetic regulators responsible for the cleavage of genes involved in the catechin biosynthetic pathway.

## 2. Materials and methods

### 2.1 Plant materials

Tea strain 1005, a tea germplasm resource with high levels of EGCG [19], was used in this study. A mixture of tea leaves, including buds, first to fifth leaves and mature leaves, were harvested from the tea garden of Fujian Agriculture and Forestry University on May 4, 2015, and used for high-throughput and degradome sequencing. First, third and mature leaves were used for expression and catechin content analyses.

### 2.2 Small RNA and degradome sequencing

Total RNA of tea strain 1005 was sequenced by Biomarker Technology Co. (Beijing, China, https://www.biocloud.net/) on an IlluminaHiSeqTM 2500 instrument. Total RNA was extracted from the strain “1005” firstly, and linkers were added to the 5'and 3' ends of RNA by T4 RNA ligase. Then target fragments were amplified by reverse transcription PCR using synthesized first-strand cDNA as template, and screened through polyacrylamide gel electrophoresis. The small RNA library was constructed by the reclaimed fragments from the gel. Biomarker Technology Company performed the construction of the small RNA library which was further processed by HiSeq2500 sequencer using single-end 50nt as sequencing read length. Bowtie software [20] was used to align the clean reads with data bases to get the unannotated sequences Which were further used to align with the miRNAs from all species in the miRBase to identify the known miRNAs of tea. Finally, to identify novel miRNAs from tea, we predicted the structure of the unannotated miRNAs and their precursors through miRDeep2 [21] and Mfold software. Structural prediction of miRNAs and their precursors was carried out using miRDeep2 and Mfold software. Gene functional annotations were performed by alignment with Gene ontology (GO) and Kyoto encyclopedia of genes and genomes (KEGG) databases.

The miRNAs used in the degradome sequencing analysis were obtained from small RNA library of cultivar 1005. After degradome sequencing, we got the clean Tags and cluster Tags by screening out the low quality ones and aligned the cluster Tags with transcriptome and Rfam database and utilized the unannotated sequences for cleavage sites analysis.

### 2.3 Quantitative real-time polymerase chain reaction analysis of miRNA expression

Total RNA was extracted by Trizol reagent (Invitrogen, USA) following the specification strictly. Using total RNA from tea leaves as template, first-strand cDNAs of miRNAs were obtained by Mir-X miRNA first-strand synthesis kit (Clontech, USA). Expression profiles of miRNAs were examined by SYBR qRT-PCR kit (Clontech, USA). And first-strand cDNAs of target genes were synthesized by Prime Script RT reagent Kit with cDNA Eraser (Takara, Jap). Expression profiles of target genes were examined using SYBR qRT-PCR kit (Takara, Jap). Primers (S1 and S2 Tables) for qRT-PCR were designed with DNAMAN software. U6 was used as a reference gene [22]. All data were generated on a Roche LightCycler 480 qPCR instrument (Roche Applied Science, Switzerland). Gene expression levels were analyzed by the 2^−ΔΔCt^ method [23].

### 2.4 Verification of microRNA targets by RNA ligase-mediated rapid amplification of 5’cDNA ends

Total RNA was separately extracted using Trizol reagent and combined in equal amounts. Total RNAs were subjected to 5’-RLM-RACE reverse transcription using a GeneRacer kit (Invitrogen, USA) following the method of Lin [24]. Total RNAs from shoots, leaves, stems and fruits of tea cultivar 1005 were ligated to a 5’ RACE RNA adapter. RNA with 5’ adapter was reverse transcribed to cDNA by using the GeneRacer OligodT primer and Superscript reverse transcriptase. Primers specific to target cleavage sites (S2 Table) were designed with DNAMAN software and used along with universal GeneRacer 5’primers for nested PCR amplifications. After separating PCR products by 1% agarose gel electrophoresis, the recovered target bands were sequenced by Huada Biotech Co.

### 2.5 High-performance liquid chromatography analysis of catechin content

We measured catechin contents of first, third and mature leaves of tea cultivar 1005. Leaf samples were dried at 120°C for 5 min followed by 60°C for 1 h. Following extraction of catechins from fully ground samples with 70% methanol in a water bath at 70°C, catechin content was measured by High-performance liquid chromatography (HPLC) at a wavelength of 278 nm using a C18 column, with three biological repeats performed per sample [25]. The catechin content measurement was performed on a Waters e2695 (Waters, England). Finally, all data were organized and analyzed using Excel 2003 and DPS software.

## 3. Results

### 3.1 Preliminary analysis of miRNA sequences from tea

**Fig 1:**
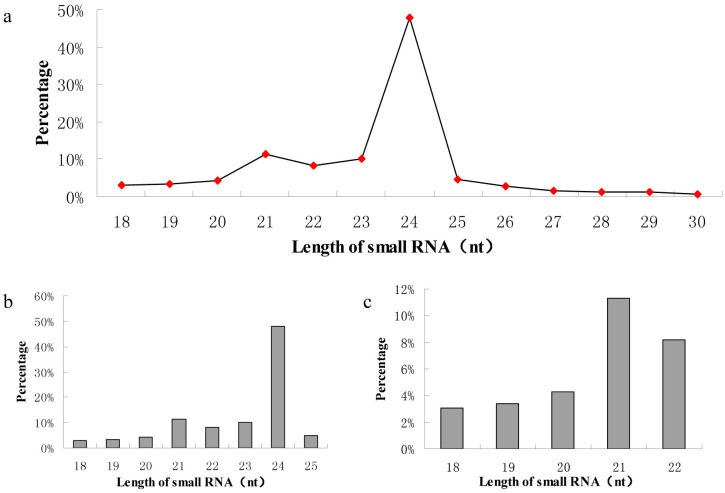
Size distribution of miRNAs by high-throughput sequencing. (a) Size distribution of ‘1005’ tea total miRNAs; (b) Size distribution of strain ‘1005’ conserved miRNAs; (c) Size distribution of strain ‘1005’ novel miRNAs.

To elucidate the roles of miRNAs in regulation of catechin biosynthesis in tea, we performed high-throughput sequencing using pooled samples of tealeaves from different maturation stages of tea cultivar 1005. After removing low-quality reads, short fragments and 3adaptor sequences, we obtained 19,567,691 clean reads. Alignment of the clean reads with various databases identified sequences corresponding to ribosomal RNA (18.83%), transfer RNA (1.23%), small nuclear RNA (0.01%) and repetitive sequences (0.07%); the remaining 79.85% were functionally unannotated and thus used in subsequent analyses to predict and identify miRNAs. According to the results of small RNA library sequencing, miRNA lengths mainly ranged from 20 to 25 nucleotides (Fig 1). Among them, the most abundant (47.89%) miRNAs consisted of 24 nucleotides, followed by 21 (11.27%) and 23 (9.97%) nucleotides. This length distribution is very similar to values reported for other tea cultivars as well as other plants [26].

### 3.2 Identification of conserved miRNAs in tea

To discover conserved miRNAs in tea cultivar 1005, we aligned the unannotated clean reads obtained above with known conserved plant miRNAs deposited in miRBase. As a result, 69 conserved miRNAs (S4 Table) classifiable into 62 families were identified. Among these families, miR160, miR396a, miR5801 and miR7519b were represented by two miRNAs each and miR426 had three members. We next analyzed the length of these conserved miRNAs. We found that they ranged from 18 to 25 nucleotides, with miRNAs containing 24 nucleotides the most abundant (46.38%), followed by those containing 21.

Analysis of the miRNA reads from high-throughput sequencing revealed that 91.3% of identified conserved miRNAs had read counts below 1,000, of which 56.52% were represented by fewer than 10 counts. We also found some highly expressed miRNA families, such as miR165a and miR396a, with read counts higher than 10,000. Although expression levels among miRNAs from different families can vary considerably, different miRNAs even within the same family showed large differences in expression. For example, members of miR426 and also of miR5801, both weakly expressed families, had different expression patterns. Similarly, read counts varied between the two members of the highly expressed miR396a family: 20,982 for one and 44,396 for the other. These data reveal differences in expression patterns of different families of miRNAs, suggesting that different miRNAs have different roles in tea growth and development.

### 3.3 Identification of novel miRNAs from tea

We identified 47 novel miRNAs (S5 Table) in tea according to common miRNA features using miRDeep2 [21] and Mfold software. We analyzed lengths and expression levels of these novel miRNAs based on the sequencing results. Similar to our findings for conserved miRNAs, these novel miRNAs ranged from 18 to 24 nucleotides in length, with 24 or 21 nucleotide-long miRNAs the most abundant (Fig 1). However, novel miRNA expression levels were lower than those of conserved miRNAs, as the highest read count obtained for a novel miRNA was only 4,517.

To look for possible homologs, we aligned the novel tea miRNAs with known miRNAs from other plants. To our surprise, this alignment revealed many homologs. Most of the miRNAs could be classified into separate families. The exception involved three miRNAs assigned to the same family, designated as novel-miR1, that were close homologs-differing by only 1 to 4 base pairs (bp)—to members of the previously known miR482 family. We thus predicted that the miRNAs in the csi-miR1 family may be new members of the miR482 family. We also analyzed the precursors of these novel miRNAs and found that they possessed a hairpin loop and ranged from 84 to 114 bps in length. The minimal folding free energy for the formation of secondary structures in the precursors ranged from 0.6 to 2.3.

### 3.4 Gene Ontology (GO) functional assignment and Kyoto Encyclopedia of Genes and Genomes (KEGG) analysis of miRNA targets

To facilitate research on the functions of tea miRNAs, we next predicted the targets of the above-identified miRNAs using TargetFinder software and transcriptome sequences (paper under review). As a result, we identified 644 target genes regulated by 97 miRNAs, with no targets determined for the other 19 miRNAs. Target genes were analyzed and categorized using GO and KEGG databases.

**Fig 2:**
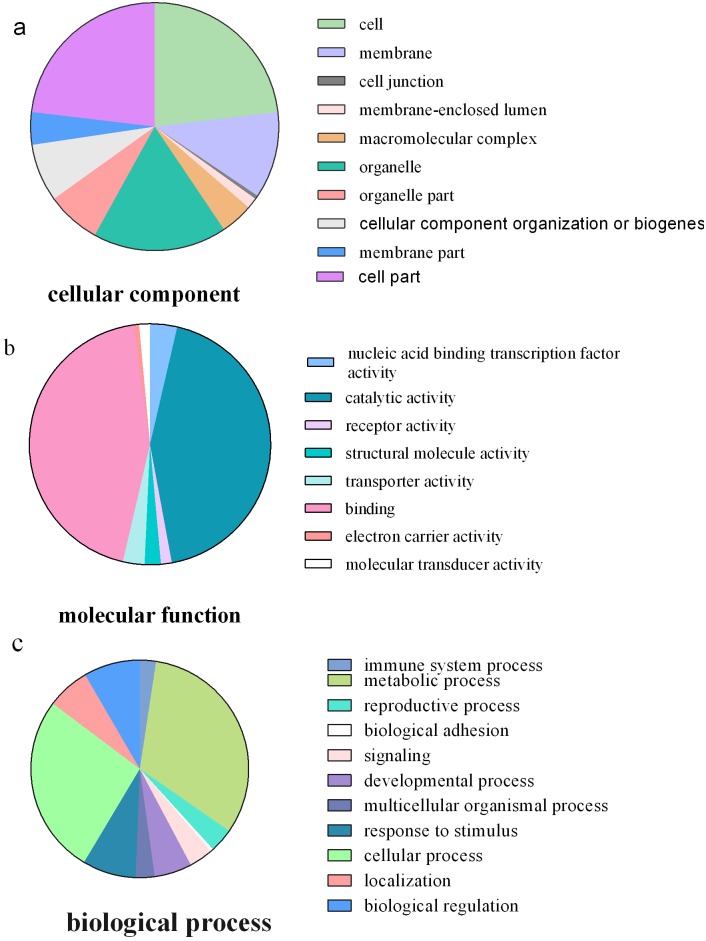
Gene ontology of the predicted targets. Categorization of target genes was accomplished according to the cellular component (a), the molecular function (b) and biological process (c).

GO categories were assigned to all target genes according to three ontologies: cellular component, molecular function and biological process (Fig 2). Functions of target genes in the cellular component category were mainly focused on cells, organelles and membranes, with some target genes related to cell junction, membrane-enclosed lumen and macromolecular complex. On the basis of molecular function, the products of more than 40% of genes were annotated as having catalytic activity. Among these, the products of 44.2% of these genes had binding activity and 2.9% had transporter activity. In regard to biological process, target genes were classified into 11 categories. Most of these genes were involved in metabolic (32.3%) and cellular (26.7%) processes, with others related to activities such as immune system, developmental and reproductive processes, biological adhesion and regulation. Some gene products were also involved in signaling pathways and responses to stimuli such as cold, hormones (abscisic acid, auxin, ethylene, jasmonic acid and salicylic acid), insects, light, salt stress, water deprivation, nitrogen starvation and pathogens (fungi and bacteria).

KEGG pathway enrichment analysis revealed that the 644 targets were involved in 36 metabolism networks. The identified metabolism networks were related to glycolysis, the citric acid cycle, amino acid metabolism, photosynthesis, fatty acid metabolism, purine and pyrimidine metabolism, oxidative phosphorylation, phytopathogen interaction, primary metabolite processes and secondary metabolic processes.

### 3.5 Degradome sequencing and functional analysis of miRNA targets regulated by conserved and novel miRNAs

To further predict and identify miRNA targets based on miRNA sequences, we constructed a degradome library and performed deep sequencing. On the basis of the transcriptome data of tea cultivar 1005 and miRNAs obtained from high-throughput sequencing, we predicted 216 targets regulated by 97 miRNAs using TargetFinder software. Of these predicted targets, degradome sequencing identified only 26 (12%) cleavage sites from 26 targets (Table 1). These 26 targets were recognized by 19 miRNAs regulating post-transcriptional expression from 16 miRNA families: csn-miR160a, csn-miR162a, csn-miR426, csn-miR4380a, csn-miR396, csn-miR394, csn-miR482, csn-miR167, csn-miR2948, csn-miR1164, novel-miR1, novel-miR2, novel-miR10, novel-miR17, novel-miR38 and novel-miR43. This phenomenon of multiple miRNA families regulating a common target has also been observed in other plant species.

**Table 1:** ‘1005’ teamiRNA targets identified by degradome sequencing.

family	miRNA	Site	Target gene annotation	Target ID
miR1164	csn-miR1164	CDS	thymidylate kinase	comp159291_c1
miR160	csn-miR160a	5'UTR	auxinresponse factor 18	comp114348_c0
		CDS	auxin response factor 18	comp157832_c1
miR162	csn-miR162a	CDS	uncharacterized protein	comp130654_c2
		CDS	endoribonucleasedicer homolog	comp163658_c0
miR167	csn-miR167a	CDS	auxin response factor 6 ARF6	comp159882_c0
		CDS	auxin response factor 8	comp157894_c0
miR2948	csn-miR2948-5p	CDS	cell division cycle protein	comp162204_c0
miR394	csn-miR394a	CDS	ADP-ribosylatiofactor GTPase-activating protein AGD12	comp156493_c0
miR396	csn-miR396a1; csn-miR396a2	CDS	DNA-directed RNA polymeraseV subunit	comp149010_c0
		CDS	calcium-transporting ATPase 13	comp163416_c1
		CDS	growth-regulating factor 7	comp152929_c0
miR426	csn-miR426a	CDS	uncharacterized protein	comp137002_c0
miR4380	csn-miR4380a	CDS	uncharacterized protein	comp142558_c0
miR482	csn-miR482b	CDS	edisease resistance protein RDL5	comp159753_c0
		CDS	disease resistance protein RGA3	comp157954_c1
miR1	novel-miR1a	3'UTR	mediatorof RNA polymeraseII transcription subunit	comp146887_c0
	novel-miR1a; novel-miR1c	CDS	uncharacterized protein	comp159862_c0
	novel-miR1a; miR1b; miR1c	CDS	V-type proton ATPase subunit	comp161699_c0
	novel-miR1b; novel-miR1c	CDS	disease resistance protein	comp162381_c0
miR2	novel-miR2	CDS	reticuline oxidase-like protein	comp135920_c0
		CDS	ethylene-responsive transcription factor 109	comp145093_c0
miR10	novel-miR10	CDS	zinc finger CCCH domain-containing protein	comp24991_c0
miR18	novel-miR18	CDS	non-specific lipid-transfer protein	comp137593_c0
miR38	novel-miR38	5'UTR	uncharacterized protein	comp138304_c0
miR43	novel-miR43	CDS	remorin	comp159672_c0

**Note:** CDS: Coding sequence; UTR: Untranslated region

In tea cultivar 1005, csn-miR160a was predicted to regulate two auxin response factor (ARF) genes which were homologous to *ARF18* of *Oryza sativa*, similar to results reported for other plants [27]. MiR167, a conserved and large family consisting of many members, exists in numerous plant species including *Arabidopsis thaliana*, *Populustrichocarpa*, *Nicotianatabacum*, *O*. *sativa* and *Zea mays* [28]. We found two target genes regulated by csn-miR167a in tea, *ARF8* and *ARF6*, which were homologs to genes in *A*. *thaliana*. The targets of several identified miRNAs, includingcsn-miR2948-5p, csn-miR162a, csn-miR394a and csn-miR482, encode cell growth and development-related proteins such as cell division cycle protein and DNA-directed RNA polymerase. Targets of some other miRNAs, such as novel-miR1 and csn-miR426, play roles in the disease resistance of tea. Two identified target genes cleaved by csn-miR482 encode products belonging to a putative disease resistance protein family; only one of these genes was found to be a homolog to the putative disease resistance protein RDL5 from *A*. *thaliana*. MiR2948, currently only found in *Gossypium hirsutum*, cleaves a target homologous to cell division cycle protein 48 from *Glycine max*. Csn-miR1164 is involved in the regulation of post-transcriptional expression of thymidylate kinase. Novel-miR1 is involved in the regulation of targets encoding homologs of proteins in *A*. *thaliana*, namely, RNA polymerase II transcription subunit 25, V-type proton ATPase subunit C, putative disease resistance protein RDL5 and a protein of unknown function. Novel-miR2 was predicted to cleave genes encoding oxidase-like protein and ethylene-responsive transcription factor (ERF), which are respectively homologous to *At4g20830*and *ERF109* from *A*. *thaliana*. Novel-miR10 regulates a target gene whose product is a zinc finger protein containing the CCCH domain. The target of novel-miR43 encodes remorin, a protein involved in the interaction between *Medicago truncatula* and *Sinorhizobium* and also interacting with multiple receptor-like kinases [29]. No other annotated miRNA targets were uncovered by degradome sequencing. Further research will be needed to fully determine the function of the targets identified in this study.

### 3.6 5’-RLM-RACE-based identification of target cleavage sites revealed by degradome sequencing

**Fig 3:**
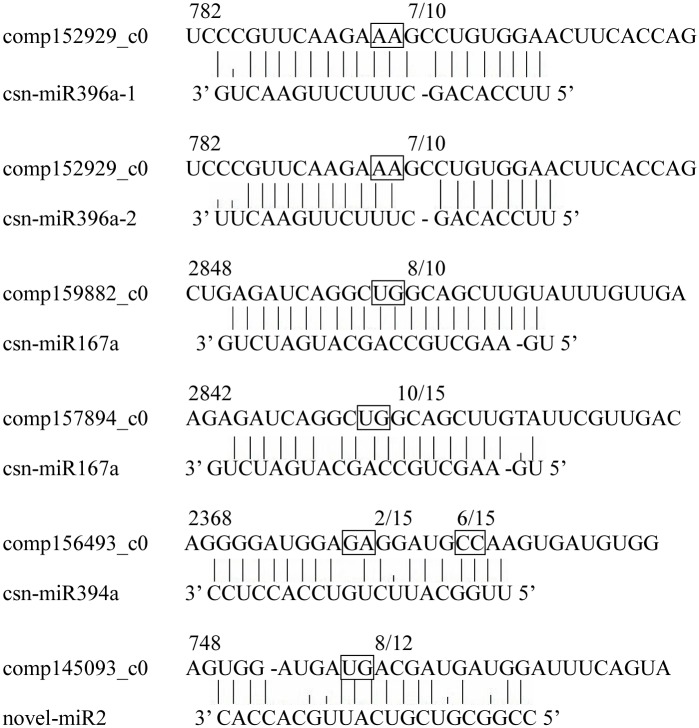
The mRNA cleavage sites of degradome sequence. Boxes indicate the cleavage sites; the numbers indicate the fraction of cloned PCR products terminating at different positions.

We used 5’-RLM-RACE to verify cleavage sites on sequences of five annotated miRNA targets from degradome sequencing (Fig 3), which partially verified the results of degradome sequencing. Using this method, we found that the target gene comp152929 is co-regulated by csn-miR396a1 and csn-miR396a2, with the cleavage site associated with miRNA binding located between the 11th and 12th nucleotides of the target. Two genes, comp159882 and comp157894, both targeted by miRNA167a, also share a cleavage site at the same location between nucleotides 11 and 12. In addition, miRNA394a may cleave comp156493 at two sites, one between nucleotides 10 and 11 and the other located between nucleotides 17 and 18. Similarly, we found that novel-miR2 may cleave comp145093 at two sites located between nucleotides 10 and 11 and between 12 and 13. Although the results of the 5’-RLM-RACE generally verified those of the degradome sequencing analysis, some cleavage sites need further verification.

### 3.7 Expression patterns of miRNAs in tea leaves

**Fig 4:**
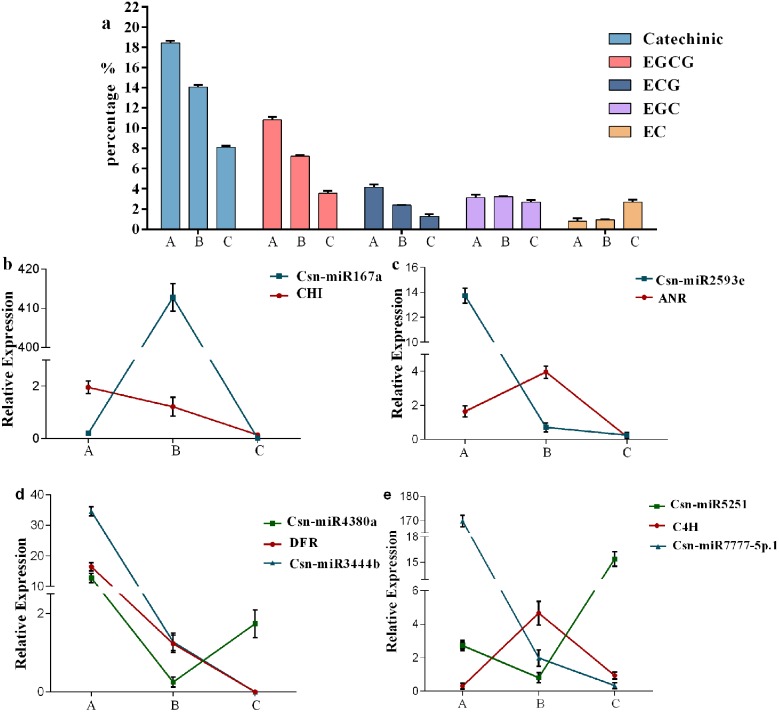
The contents of catechin and expression levels of miRNAs and target genes in tea leaves of different maturity. A, first leaves; B, third leaves; C, old leaves. The X axis in Fig 4 indicates the position of leaves; the Y axis in Fig 4a indicates percentages of catechin in dry tea leaves; the Y axis in Fig 4b-e indicates relative expression.

**Fig 5:**
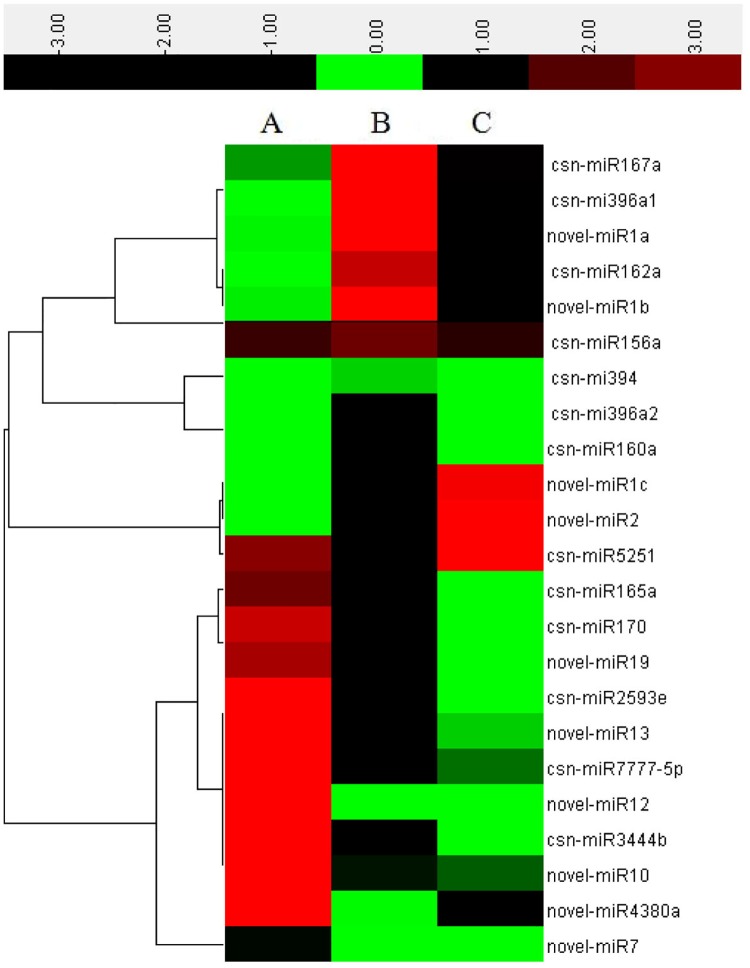
Expression of miRNAs at different levels of maturity. A, first leaves; B, third leaves; C, old leaves.

In previous research on the catechin content of different leaf types of tea cultivar 1005, we found that catechins were most abundant in first leaves, followed by buds, second leaves and third leaves, with the lowest catechin content in mature leaves. Catechin contents of first, third and mature leaves were significantly different from one another (Fig 4a). To uncover miRNAs related to catechin synthesis in tea, we used quantitative real-time PCR (qRT-PCR) to analyze the expression levels of miRNAs having high-throughput sequencing read values higher than average in first, third and mature leaves from tea cultivar 1005. These three types of leaves were chosen because they had markedly different catechin contents (Fig 5). Cluster analysis of the expression patterns divided these miRNAs into three groups. The first group comprised novel-miR10, novel-miR13, novel-miR19, csn-miR165a, csn-miR170, csn-miR2593e and novel-miR12; the expression patterns of these miRNAs were positively correlated with the pattern of catechin content, being most highly expressed in first leaves, followed by third leaves and then mature leaves. miRNAs in the second group, which consisted of novel-miR1, novel-miR2, csn-miR160a, csn-miR162a, csn-miR394 and csn-miR396a families, all dramatically fluctuated in the same manner among first, third and mature leaves. They all had their highest expressions in third leaves followed by first leaves, with no expression detected in mature leaves. This pattern suggests that the expressions of these miRNAs are negatively correlated with catechin content. The third group, composed solely of csn-miR4380a, showed no obvious correlation between expression and catechin content: the highest expression was observed in first leaves and the lowest in third leaves.

### 3.8 Identification of miRNAs regulating catechin biosynthetic pathway genes

**Fig 6:**
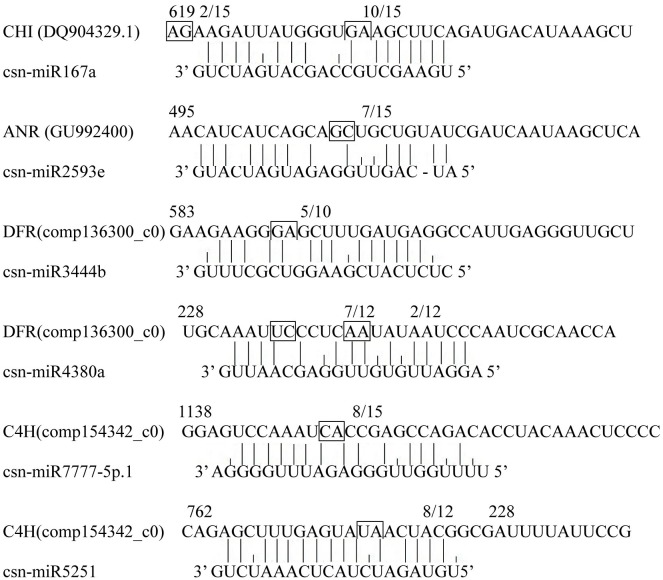
The mRNA cleavage sites identified by 5’RLM-RACE. Boxes indicate the cleavage sites; the numbers indicate the fraction of cloned PCR products terminating at different positions.

To further understand the function of miRNAs in the regulation of catechin anabolism, we predicted potential miRNAs by subjecting miRNAs obtained from high-throughput sequencing to a BLAST search against catechin biosynthesis-related gene mRNAs from National Center for Biotechnology Information (NCBI) database and RNA sequencing (S6 Table). To eliminate false positives, the predicted cleavage sites were then experimentally verified. 5’-RLM-RACE experiments confirmed that six potential miRNAs are involved in regulating the expression of genes functioning in the catechin biosynthetic pathway (Fig 6). CHI was verified as a target of csn-miRNA167a, which regulates the expression of a CHI-encoded gene by cleaving the gene between the 13th and 14th nucleotides of the binding area. ANR was verified as a target of csn-miR2593e. ANR can be regulated by csn-miR2593e by cleavage in the binding region between the 12th and 13th nucleotides. Both CHI mRNA and ANR mRNA were found to harbor only one cleavage site. In the biosynthesis of all classes of flavonoids, CHI is a key early-stage enzyme [30], while the enzyme ANR controls catechin and procyanidin biosynthesis. The discovery of these cleavage sites suggests that csn-miR2593e and csn-miRNA167a play major regulatory roles in the flavonoid biosynthetic pathway. Some target mRNAs were determined to have multiple cleavage sites, similar to Arabidopsis [30]. According to our experiments, DFR was cleaved at positions 236, 242 and 592. Sites at positions 236 and 242 were cleaved by csn-miR4380a, while a site at position 592 was the target of csn-miR3444b. C4H was cleaved by csn-miR5251 and csn-miR7777-5p.1 at positions 777 and 1,150, respectively. During this verification, we also found some potential cleavage sites in target genes, which suggested that some other unknown miRNAs may function in the regulation of catechin synthesis. These results thus reveal that miRNAs indeed play crucial regulatory roles in the catechin biosynthetic pathway. More research is needed to identify other potential miRNAs involved in regulation of catechin biosynthesis.

### 3.9 Expression analysis of catechin biosynthetic pathway genes and miRNAs

To better understand expression patterns and interactions of miRNAs and target genes during catechin biosynthesis, the expressions of catechin biosynthesis-related genes and miRNAs in different leaves were analyzed and compared with the pattern of variation in catechin content (Fig 4). ANR and C4H had similar expression patterns. Expression levels of ANR, which were highest in third leaves, showed no obvious correlation with EC, EGC or catechin contents. This result is consistent with that reported for Shuchazao green tea [31]. CHI and DFR expression levels and total catechin content all followed the same trend, a result in accord with a previous report [32]. According to our analysis, expression patterns of most miRNAs displayed an opposite tendency to that of their targets with a few exceptions. For example, DFR was targeted by both csn-miR3444b and csn-miR4380a, but only csn-miR3444b had an expression trend similar to that of DFR in different tea leaves. In particular, csn-miR4380a and DFR had their highest expressions in first leaves, whereas csn-miR4380a was negatively correlated with DFR in third and mature leaves. In summary, the negative correlation in expression patterns and cleavage sites observed between miRNAs and target mRNAs is evidence that miRNAs target mRNAs, with regulatory roles suggested for miRNAs in catechin biosynthesis.

## 4. Discussion

### 4.1 Identification of miRNAs and their targets through high-throughput and degradome sequencing

In this study, sequencing of a miRNA library constructed from tea cultivar 1005 yielded 69 conserved and 47 novel miRNAs, thereby enlarging the number of known tea miRNAs and their categories. In a previous investigation, Zhang Yue identified 106 conserved and 98 novel miRNAs from tea cultivars Yingshuang and Baiye under different cold-stress treatments by high-throughput sequencing [33]. As differences in the accumulation of miRNAs are apparent between different varieties and treatment conditions, sequencing of plant samples from different tea varieties under various treatments will facilitate the discovery of more miRNAs in tea. Before our work, Akan Das identified 13 conserved miRNAs that can be classified into 9 families by bioinformatics method and Quanwu Zhu found 14 conserved miRNAs from 9 families through expressed sequence tag (EST) analysis [34]. However, when aligning these previously identified miRNAs with ours, we found that only partial was comparable. One possible reason is that the bioinformatics method only confirmed the existence but not the sequences of the miRNAs. Another possible reason is the loss of some potential miRNAs during data analysis due to the uncompleted EST and genome information of tea. In addition, we note that more than 80% of miRNAs obtained from our high-throughput sequencing data were unannotated, which indicates that much more work is needed to identify novel miRNAs.

Degradome sequencing can be used to identify the relationship between miRNAs and their targets. Since 2008, this method has been applied to identify targets of miRNAs in many plant species, such as *A*. *thaliana* [35], *Vitis vinifera* [36], *Z*. *mays* [37] and *Brassica rapa* ssp. *pekinensis* [38]. In the study, we identified 26 targets regulated by 16 miRNA families. Most of these targets were homologous to genes from *A*. *thaliana*. Some of the identified targets encode proteins involved in the regulation of plant growth and development, including ARF, ERF, growth-regulating factor (GRF) and zinc finger CCCH-domain-containing protein. We identified some targets of tea miRNAs, which should aid research on the function of miRNAs and targets. Nevertheless, only a small fraction of targets were revealed by degradome sequencing; identification of the majority of miRNAs and targets will require further effort.

### 4.2 Functions of miRNAs in the regulation of catechin biosynthesis

Tea, a globally popular, healthful beverage, confers anti-oxidative benefits due to the presence of abundant polyphenols having anti-aging and anti-cancer properties [9]. Catechin is one of the most important polyphenols in tea. Researchers from Anhui Agricultural University have already worked out the synthetic pathways of ester-type catechins, thus facilitating further research on the elucidation of catechin metabolism in tea [11–13]. The results of degradome sequencing showed that both csn-miR160a and csn-miR167 regulated ARF family proteins, such as *ARF6*, *ARF8* and *ARF18*, which also expressed in many other plant species [39], including *A*. *thaliana* [40], *Glycine max* [41] and *Euphoria longan* [42]. According to the previous report, *ARF18*, similar to *ARF1* and *ARF2*, is a transcriptional repressor in ARF family [43]. In rice, miR160a controls etiolation of leaves by cleaving *ARF18* [44]. The expressions of *ARF6* and *ARF8* are negatively regulated by miR167a, thereby influencing flower and fruit growth and development in *A*. *thaliana* [45, 46]. Over-expression of miR160a suppresses the expression of target ARF genes and negatively controls leaf senescence in *Glycine max* [41]. Conserved miRNA csn-miR396 regulates *csiGRF*, homologous to *AtGRF7*, which was one of the GRF family in *A*. *Thaliana* and also regulated by miR396 [47, 48]. *AtGRF7* was reported to function as a transcriptional repressor of genes responding to abscisic acid and osmotic stress [47]. GRF family proteins appear to promote and maintain cell proliferation activity in leaves since evidences have shown that miR396 negatively regulated cell proliferation by repressing *GRF* expression in *A*. *thaliana* [49]. In addition, miR396 controls leaf size by regulating *GRF* genes in *A*. *thaliana* [47, 48], for example, over-expression of miR396 or mutation of *GRFs* leads to smaller leaves in *A*. *thaliana*, in contrast, leaves are enlarged when the binding sitesin*GRF7* or *GRF9* for miR396 are mutated, these data indicate that miR396 and its targets are involved in the regulation of leaf growth and development [47]. Generally, csn-miR160a, csn-miR167a and csn-miR396a all play essential roles in leaf development by regulating target genes. On the other hand, expression analysis uncovered several miRNAs, includingcsn-miR160a, csn-miR167a and csn-miR396a, which negatively regulate catechin biosynthesis. Moreover, previous research on flavonoids indicates that phenolic compounds, especially catechins, are most abundant in younger leaves [50]. Catechin biosynthesis is thus likely influenced by csn-miR160a, csn-miR167a and csn-miR396a, which target genes that regulate leaf development in tea plant.

5’-RLM-RACE results indicate that CHI, a key enzyme converting chalcone into flavones during flavonoid synthesis [51], is targeted by csn-miR167a. Some research indicates that over-expression of *CHI* slightly increases the flavonoid content of pulp and pericarp in tomato [52]. We observed that catechin content was negatively correlated with the expression of csn-miR167a, which directly cleaves the *CHI* gene. We thus conclude that csn-miR167a regulation of catechin biosynthesis involves cleavage of *CHI* mRNA. We also performed 5’-RLM-RACE assays on *C4H*targetedby csn-miR5251 and csn-miR7777-5P.1. C4H, a key enzyme of the phenylalanine metabolic pathway, provides precursor substances for secondary metabolites such as lignins and flavonoids [53]. *C4H* inactivation has been found to reduce the accumulation of flavonoids in *A*. *thaliana* [54]. Our results indicate that csn-miR5251 targets *C4H*, with the expression of csn-miR5251 negatively correlated with that of *C4H*. In contrast, however, some researchers have indicated that miR5251 targets leucoanthocyanidin dioxygenase in *Carthamus tinctorius*. In that study, over-expression of the pre-miR5251 of *Carthamu stinctorius*in *A*. *thaliana* caused an increase in the flavonoid contents of leaves, stems and roots [55]. These contradictory results may be due to diversity in regulatory functions and target genes of miRNAs among different species or alternatively be a consequence of different miR5251 members being verified. Our analysis demonstrated that csn-miR5251 and csn-miR7777-5P.1 function as negative regulators of gene expression by managing the cleavage of *C4H* mRNA. *C4H* expression shared a similar trend with catechin content, a result consistent with the findings of another study [56]. This observation suggests that csn-miR5251 and csn-miR7777-5P.1 influence catechin biosynthesis by mediating *C4H* mRNA. On the other hand, some MYB transcription factors have been found to negatively regulate *C4H* expression in *A*. *thaliana* [57] and *N*. *tabacum* [58]. In addition, *C4H* is considered to be a target gene of *CsMYB4-4* and *CsMYB4-6* in tea [59]. We thus speculate that *C4H* transcription is concurrently regulated by miRNAs and transcription factors.

DFR is a downstream enzyme of flavonoid metabolism and a key enzyme of catechin biosynthesis [60]. *DFR* expression levels have been found to be correlated with catechin content [61, 62]. Our results indicate that *DFR* is regulated by csn-miR4380a and csn-miR3444b. Only a few studies have addressed the direct relationship between miRNAs and targeted DFR. According to one study, *DFR* expression is controlled by miR156-SPL9-DFR in *A*. *thaliana* [63]. In addition, some studies of tea have revealed a possible mechanism of csn-miR156-mediated regulation of catechin biosynthesis, namely, that csn-miR156 regulates *DFR* transcription by controlling *SPL* expression to influence catechin biosynthesis. This function for csn-miR156 is consistent with findings in *A*. *thaliana* [32].

By the way, research in plants has revealed that many miRNAs play essential roles in plant development and influence flavonoid metabolism by regulating transcription factors. For example, miR828 controls anthocyanin biosynthesis by targeting MYB7-like in Lycopersiconesculentum [64], while miR858 targets AtMYB12 to regulate flavonoid biosynthesis in A. thaliana [65]. These studies further explain that miRNAs clearly play important roles in flavonoid metabolism at transcriptional and post transcriptional levels.

**Fig 7:**
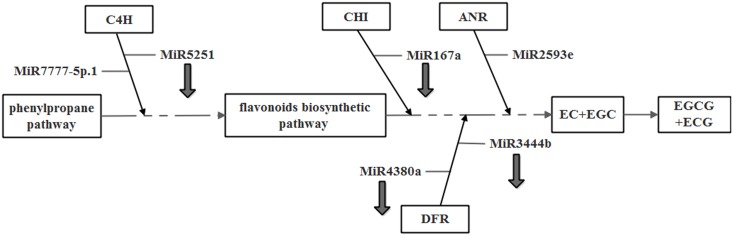
The potential patterns of miRNAs that regulated cetechins synthesis in tea cultivar 1005. The arrows pointing down represent down-regulating on catechins content.

In summary, combining the miRNA library and catechins biosynthetic pathway genes which have been cloned, the potential miRNAs were predicted. Through 5’-RLM-RACE and expression analysis, six potential miRNAs were proved to cleave targets and might be involved in regulating the expression of target genes (Fig 7). These results revealed that miRNAs indeed played important roles in the of catechins anabolism, and also provide information for the regulation of catechins biosynthesis.

## Supporting information

S1 Table Specific primers used for quantitative real-time PCR of miRNAs. (DOC)

S2 Table Specific primers used for quantitative real-time PCR of target genes. (DOC)

S3 Table Oligonucleotide primers used for the miRNA cleavage sites. (DOC)

S4 Table Mature sequences and counts of conserved miRNAs in Small RNA sequencing. (DOC)

S5 Table Mature sequences and counts of novel miRNAs in Small RNA sequencing. (DOC)

S6 Table Potential miRNAs regulating catechins biosynthetic pathway genes. (DOC)

S1 Appendix A list of Abbreviations used in this manuscript. (DOC)
