# Supplementary material for: Combined small RNA and degradome sequencing reveals complex microRNA regulation of catechin biosynthesis in tea (Camellia sinensis)
Source: PLoS One. 2017 Feb 22;12(2):e0171173. doi: 10.1371/journal.pone.0171173 (PMC5321428; doi:10.1371/journal.pone.0171173)
Supplement: S1 Table — (DOC) [file pone.0171173.s001.doc]

**S1 Table. Specific primers used for quantitative real-time PCR of miRNAs**

| **MiRNA** | **Primer sequences 5’→3’** | **Temperatures (℃)** |
| --- | --- | --- |
| Novel-miR1a | TCCAAGTCCACCCATTCC | 59 |
| Novel-miR1b | CCTAAACCACCCATTCCTC | 58 |
| Novel-miR1c | TTCCAAGACCACCCATGC | 60 |
| Novel-miR2 | GGCGTCGTCATTGCACC | 61 |
| Novel-miR7 | GTGCTGTCTATCGTCGTCAT | 55 |
| Novel-miR10 | CGCAGAAGAGATGACACTTG | 58 |
| Novel-miR12 | TGACAGCAAATGCUCATG | 57 |
| Novel-miR13 | CGGATTGGTTTCGGATTG | 59 |
| Novel-miR19 | GAGAACCATGGGGAGATG | 59 |
| Csn-miR167a | GAAGCTGCCAGCATGATC | 58 |
| Csn-miR162a | TCGATAAACCTCTGCATCC | 57 |
| Csn-miR156a | GGAGAGAGAGAGAGAGAGAGC | 58 |
| Csn-miR160a | TGCCTGGCTCCCTGTATG | 60 |
| Csn-miR165a | TCGGACCAGGCTTCATTC | 60 |
| Csn-miR170 | TGAGCCGTGCCAATATC | 59 |
| Csn-miR396a1 | TTCCACAGCTTTCTTGAACTG | 58 |
| Csn-miR396a2 | GCCACAGCTTTCTTGAACTT | 57 |
| Csn-miR394 | TGGCATTCTGTCCACCTC | 58 |
| Csn-miR2593e | GCGTTGGAGATGATCATG | 59 |
| Csn-miR5251 | GCGATCTACTCAAATCTGATG | 55 |
| Csn-miR4380a | CGAGGATTGTGTTGGAGC | 59 |
| Csn-miR7777-5p.1 | TGGTTGGGAGATTTGGG | 62 |
| Csn-miR3444b | GCTCTCATCGAAGGTCGC | 62 |
