# Supplementary material for: Combined small RNA and degradome sequencing reveals complex microRNA regulation of catechin biosynthesis in tea (Camellia sinensis)
Source: PLoS One. 2017 Feb 22;12(2):e0171173. doi: 10.1371/journal.pone.0171173 (PMC5321428; doi:10.1371/journal.pone.0171173)
Supplement: S2 Table — (DOC) [file pone.0171173.s002.doc]

**S2 Table.Specific primers used for quantitative real-time PCR of target genes**

| Gene name | Primer sequences | Product size (bp) | Temperatures (℃) |
| --- | --- | --- | --- |
| Anthocyanidin reductase (ANR) | F: TTGTGGCAGAGAAAGAATCGG | 200 | 60 |
|  | R: CCCATACTTGAAACTGAATCCCTC |  |  |
| Chalcone isomerase (CHI) | F: TCTCTCTCCTAAACTCTCATCG | 175 | 59 |
|  | R: CATTTGTGGCTCTTCATCAG |  |  |
| Dihydroflavonol 4-reductase (DFR) | F: GATTCATCGGCTCGTGG | 138 | 61 |
|  | R: GGAAGGCGGATTTGAATG |  |  |
| Cinnamate 4- hydroxylase (C4H) | F: AACCACCGCAACTTGACC | 221 | 60 |
|  | R: CCTCCAGTGCTCACCGT |  |  |
