# Supplementary material for: Combined small RNA and degradome sequencing reveals complex microRNA regulation of catechin biosynthesis in tea (Camellia sinensis)
Source: PLoS One. 2017 Feb 22;12(2):e0171173. doi: 10.1371/journal.pone.0171173 (PMC5321428; doi:10.1371/journal.pone.0171173)
Supplement: S3 Table — (DOC) [file pone.0171173.s003.doc]

**S3 Table. Oligonucleotide primers used for the miRNA cleavage sites**

| **miRNA** | **Targets ID** | **Target gene annotation** | **Gene specific primer** | **Primer sequences(5’-3’)** |
| --- | --- | --- | --- | --- |
| Csn-miR2 | comp145093-c0 | Ethylene responsive transcription factor 109 | Gene-specific primer | GAACTCTGAGCATTTCCAGTG |
|  |  |  | Nest specific primer | GACAATTCTGATTCCGGGTC |
| Csn-miR167a | comp159882-c0 | Auxin response factor 6 | Gene-specific primer | TCCTGTCCACTTGCGTAGTC |
|  |  |  | Nest specific primer | AGCCTCTGTGTTGGGACC |
| Csn-miR167a | comp157894-c0 | Auxin response factor 8 | Gene-specific primer | GCCCAGAAACAAGGTCTCG |
|  |  |  | Nest specific primer | TCGTTCCCGCTGGTATTT |
| Csn-miR396a-1 | comp152929-c0 | Growth-regulating factor 7 | Gene-specific primer | CCACCATCATACCACCCG |
|  |  |  | Nest specific primer | GGATGAGAATGGTTGGGTCC |
| Csn-miR396a-2 | comp152929-c0 | Growth-regulating factor 7 | Gene-specific primer | CCACCATCATACCACCCG |
|  |  |  | Nest specific primer | GGATGAGAATGGTTGGGTCC |
| Csn-miR394 | comp156493-c0 | ADP-ribosylation factor GTPase-activating | Gene-specific primer | AGAACGACATATGCGGGC |
|  |  |  | Nest specific primer | TCTTTCATGGGCATGCC |
| Csn-miR167a | DQ904329.1 | Chalcone isomerase (CHI) | Gene-specific primer | CATCATTGCCGAGAAACAGT |
|  |  |  | Nest specific primer | GGAAACCAACCACTCTCCTG |
| Csn-miR2593e | GU992400 | Anthocyanidin reductase (ANR) | Gene-specific primer | GGGAGATGGAGATTGAGCCT |
|  |  |  | Nest specific primer | GAGAGTTGGGATGACAGTGAT |
| Csn-miR3444b | Comp136300_c0 | Dihydroflavonol 4-reductase (DFR) | Gene-specific primer | GCTGCTTTCTCTGCCAATG |
|  |  |  | Nest specific primer | AACGGGTTGTTGGTGTTCC |
| Csn-miR4380a | Comp136300_c0 | Dihydroflavonol 4-reductase (DFR) | Gene-specific primer | CCTTCTTCATTCAAATCCGC |
|  |  |  | Nest specific primer | GCTTTCGGCAAGTCTAACAAG |
| Csn-miR7777-5p.1 | Comp154342_c0 | Cinnamate 4- hydroxylase (C4H) | Gene-specific primer | CAGTTGTCAGGGTTGTTTGC |
|  |  |  | Nest specific primer | CAGCAGGGATGTCATAGCC |
| Csn-miR5251 | Comp154342_c0 | Cinnamate 4- hydroxylase (C4H) | Gene-specific primer | CAGCAGGGATGTCATAGCC |
|  |  |  | Nest specific primer | GTTTGTAGGTGTCTGGCTCG |
