# Supplementary material for: Combined small RNA and degradome sequencing reveals complex microRNA regulation of catechin biosynthesis in tea (Camellia sinensis)
Source: PLoS One. 2017 Feb 22;12(2):e0171173. doi: 10.1371/journal.pone.0171173 (PMC5321428; doi:10.1371/journal.pone.0171173)
Supplement: S4 Table — (DOC) [file pone.0171173.s004.doc]

**S4 Table. Mature sequences and counts of conserved miRNAs in Small RNA sequencing**

| **ID** | **MiRNA** | **Mature sequence** | **Counts** | **Length** |
| --- | --- | --- | --- | --- |
| conservative_comp97221_c0_92972 | ath-miR165a | ucggaccaggcuucauucccc | 115491 | 21 |
| conservative_comp96227_c0_210116 | ath-miR170 | ugauugagccgugccaauauc | 904 | 21 |
| conservative_comp95877_c0_16210 | ath-miR3440b-5p | auuucuugguugcuuuacucc | 915 | 21 |
| conservative_comp89234_c0_99287 | ptc-miR6455 | gcaaauaggauauaugggcagagu | 2 | 24 |
| conservative_comp7846_c0_53682 | ptc-miR6443 | cuaugaucuaugguaacauguaau | 22 | 24 |
| conservative_comp774352_c0_106652 | sbi-miR5381 | uagaucugaggcugacuggcucuc | 1 | 24 |
| conservative_comp73028_c0_132548 | ath-miR2933a | aaaaucggucuugagcucagcucg | 27 | 24 |
| conservative_comp55881_c0_209579 | stu-miR7985 | agggcuugagccaucguuuugaag | 4 | 24 |
| conservative_comp4599877_c0_201380 | bdi-miR7775-5p | accgguuuguguaaauugaucaac | 5 | 24 |
| conservative_comp43252_c0_49437 | osa-miR159a.2 | cugcaugcuugauggaggug | 2 | 20 |
| conservative_comp3610748_c0_166107 | bdi-miR7777-5p.1 | uuuugguugggagauuugggga | 11 | 22 |
| conservative_comp33672_c0_28317 | mtr-miR2664a | aauuguggccuuuagaucaagugu | 49 | 24 |
| conservative_comp33511_c0_29862 | tae-miR1127 | uccuuccguuggaacaggcgaugu | 2 | 24 |
| conservative_comp1828432_c0_303379 | mtr-miR2641 | auuugaucuuggcccuugaugugu | 59 | 24 |
| conservative_comp163697_c0_171286 | aly-miR868-5p | cuuucucggaggaauucuuug | 117 | 21 |
| conservative_comp162723_c0_95360 | ath-miR156a | ugacagaagagagugagcaca | 887 | 21 |
| conservative_comp162107_c1_118705 | ppt-miR477h | acucccuccgucccaaaauaaugg | 9 | 24 |
| conservative_comp161929_c0_217386 | osa-miR5498 | agagcuguuuuguugcuuccaugu | 4 | 24 |
| conservative_comp161244_c1_12387 | vvi-miR3629a-3p | agcugcuguggugccacugauggu | 11 | 24 |
| conservative_comp160152_c0_165883 | cre-miR918 | caccugaaccuuuugaaccuggcc | 4 | 24 |
| conservative_comp160027_c3_185959 | osa-miR1441 | cccggauggaaaaacuauu | 1 | 19 |
| conservative_comp159798_c0_170160 | aly-miR399d-5p | aggcgaauucggacuaaacaaauu | 2 | 24 |
| conservative_comp159729_c1_278108 | ath-miR401 | ugaaacuguggcuccggucgau | 12 | 22 |
| conservative_comp159355_c0_321659 | gma-miR4380a | aggauuguguuggagcaauugaug | 3 | 24 |
| conservative_comp158556_c0_22797 | lja-miR7519 | aaaauuuuuuuagucgguaaaagc | 5 | 24 |
| conservative_comp158556_c0_22795 | lja-miR7519 | aaaauuuuuuuagucgguaaaagc | 5 | 24 |
| conservative_comp158453_c2_196519 | aly-miR4232 | ucacauuucaccuaccuuuucc | 13 | 22 |
| conservative_comp158069_c2_133299 | ghr-miR2948-5p | ggugggagcuccgugaauucgccua | 2 | 25 |
| conservative_comp156658_c3_74281 | ath-miR426 | auuuggaaagagaaauggaaa | 160 | 21 |
| conservative_comp156563_c0_348063 | sbi-miR6217a-5p | aagccacugugguugagccugugu | 1 | 24 |
| conservative_comp155867_c2_149808 | mtr-miR5251 | uguagaucuacucaaaucugaugu | 7 | 24 |
| conservative_comp155840_c0_247521 | ath-miR426 | auuuggaaaaggaaagagaaaag | 63 | 23 |
| conservative_comp154857_c3_94319 | csi-miR482b | ucuugcccagaccucccauacc | 237 | 22 |
| conservative_comp152024_c0_7164 | aly-miR3444b | cucucaucgaaggucgcuuug | 2 | 21 |
| conservative_comp151936_c1_120735 | pab-miR950-3p | ccugggccaucguuuugaagc | 3 | 21 |
| conservative_comp151679_c0_77781 | cre-miR1165-3p | acggaccgacuucaccuuauaagc | 3 | 24 |
| conservative_comp151144_c0_234621 | bdi-miR7785-3p | auucuucuagaucugcucuaaauc | 2 | 24 |
| conservative_comp150889_c0_330111 | ath-miR400 | uaugagaggauuguauuuagu | 2 | 21 |
| conservative_comp148755_c0_347435 | mtr-miR2593e | auacaucaguuggagaugaucaug | 4 | 24 |
| conservative_comp148265_c0_285156 | bdi-miR397b-3p | auucgacccucugauauuucgagc | 115 | 24 |
| conservative_comp148230_c0_278888 | bdi-miR5181a-5p | cgauccauggaucgucgacug | 3 | 21 |
| conservative_comp1473952_c0_155449 | aly-miR160c-3p | ucguacaaaaguuguuuggcuau | 2 | 23 |
| conservative_comp147207_c0_100591 | cre-miR1164 | uggugcaagcgguccugugcucu | 4 | 23 |
| conservative_comp146532_c1_91896 | osa-miR5801 | uccaaaucccuccccaaauc | 28 | 20 |
| conservative_comp145684_c1_312479 | ath-miR394a | uuggcauucuguccaccucc | 2582 | 20 |
| conservative_comp144547_c0_115671 | osa-miR5801 | uccaaaucguuguucagagagaga | 1 | 24 |
| conservative_comp143751_c0_433 | aly-miR851-3p | aguggguguauccggaccggaacc | 5 | 24 |
| conservative_comp142298_c0_235554 | mtr-miR1510b-5p | gcauggaugugggauucacauggg | 2 | 24 |
| conservative_comp140003_c1_124157 | ath-miR396a | uuccacagcuuucuugaacuu | 44396 | 21 |
| conservative_comp137684_c0_48119 | osa-miR5543 | caugaaugaugcgggagauag | 7 | 21 |
| conservative_comp135691_c0_176424 | osa-miR2097-5p | ugagaugguuguguccgaugc | 68 | 21 |
| conservative_comp134570_c0_312175 | bdi-miR7741-3p.1 | agaucuucuucgcgacgaggcgug | 2 | 24 |
| conservative_comp133813_c0_16719 | bdi-miR7717c-3p | uuaguugagauucauucugagugu | 33 | 24 |
| conservative_comp132049_c0_350461 | mtr-miR2680a | accucggucucuggaccaugaccg | 1 | 24 |
| conservative_comp131494_c0_253494 | ath-miR162a | ucgauaaaccucugcauccag | 7303 | 21 |
| conservative_comp130814_c0_45905 | ath-miR160a | ugccuggcucccuguaugcca | 52 | 21 |
| conservative_comp125157_c0_246984 | mtr-miR2633 | agacauuugguagaauuuu | 18 | 19 |
| conservative_comp124269_c0_160297 | ath-miR396a | uuccacagcuuucuugaacug | 20982 | 21 |
| conservative_comp119186_c0_174475 | ath-miR426 | auuuggaaagagaaagga | 3 | 18 |
| conservative_comp118024_c0_121321 | osa-miR5080 | uaaaggauagcucugcagggcgu | 2 | 23 |
| conservative_comp1163_c1_8371 | ath-miR167a | ugaagcugccagcaugaucug | 6727 | 21 |
| conservative_comp115541_c0_163137 | hvu-miR6212 | cuacaguugggaaaggauaagacu | 5 | 24 |
| conservative_comp113862_c0_107016 | aly-miR4241 | auuugggaagggauuuagaaacgg | 18 | 24 |
| conservative_comp113213_c0_170395 | mtr-miR5272a | aaauugauucuggaccacacauc | 2 | 23 |
| conservative_comp111134_c0_259012 | gma-miR4998 | uguuucguugaguuuaaug | 3 | 19 |
| conservative_comp109921_c1_214192 | gma-miR1520e | caauaagauucaaggagacag | 15 | 21 |
| conservative_comp109792_c0_20679 | ath-miR5023 | uuugguagaaaauuugggacc | 64 | 21 |
| conservative_comp1072511_c0_94087 | ptc-miR1450 | aucaauggcuggcaaacaagaagu | 2 | 24 |
| conservative_comp104219_c0_56418 | ghr-miR7497 | ccaugugguggcugauauuu | 5 | 20 |
