# Supplementary material for: Combined small RNA and degradome sequencing reveals complex microRNA regulation of catechin biosynthesis in tea (Camellia sinensis)
Source: PLoS One. 2017 Feb 22;12(2):e0171173. doi: 10.1371/journal.pone.0171173 (PMC5321428; doi:10.1371/journal.pone.0171173)
Supplement: S5 Table — (DOC) [file pone.0171173.s005.doc]

**S5 Table. Mature sequences and counts of novel miRNAs in Small RNA sequencing**

| **MiRNA** | **ID** | **Mature sequence** | **Counts** | **Length** |
| --- | --- | --- | --- | --- |
| novel-miR1a | unconservative_comp136449_c0_282484 | uuuccaaguccacccauuccua | 10659 | 22 |
| novel-miR1b | unconservative_comp135682_c0_35812 | uuuccuaaaccacccauuccuc | 3944 | 22 |
| novel-miR1c | unconservative_comp158118_c5_196002 | uuuccaagaccacccaugccga | 2370 | 22 |
| novel-miR2 | unconservative_comp23573_c0_25881 | ccggcgucgucauugcaccac | 4507 | 21 |
| novel-miR4 | unconservative_comp161085_c0_107831 | aaugcucugauaccauguuaaagu | 2749 | 24 |
| novel-miR6 | unconservative_comp120417_c0_48248 | acugacguguucaacuugaauugg | 1988 | 24 |
| novel-miR7 | unconservative_comp129383_c0_344894 | gugcugucuaucgucgucaug | 330 | 21 |
| novel-miR8 | unconservative_comp1332541_c0_177937 | uaaaugcgaucccuugggaau | 249 | 21 |
| novel-miR9 | unconservative_comp113245_c0_254175 | aaguuaguuuguuuggcauauucu | 70 | 24 |
| novel-miR10 | unconservative_comp111176_c0_298581 | ucgcagaagagaugacacuugg | 70 | 22 |
| novel-miR11 | unconservative_comp28970_c0_76539 | auagugugacuggacuguuagucc | 77 | 24 |
| novel-miR12 | unconservative_comp1917388_c0_292420 | auggccugacagcaaaugcucaug | 66 | 24 |
| novel-miR13 | unconservative_comp101331_c0_163583 | acggauugguuucggauugaaccc | 90 | 24 |
| novel-miR14 | unconservative_comp159467_c2_117679 | uucccuaauugaugucggacaauu | 120 | 24 |
| novel-miR15 | unconservative_comp159095_c0_96677 | acgauacucacaugcguguggaac | 72 | 24 |
| novel-miR16 | unconservative_comp163773_c0_90336 | auuucggaugcugcaucagacggu | 44 | 24 |
| novel-miR18 | unconservative_comp963337_c0_191700 | cucacaaguggacuccaauug | 27 | 21 |
| novel-miR19 | unconservative_comp7014_c0_148291 | uacuuggagaaccauggggagaug | 94 | 24 |
| novel-miR20 | unconservative_comp155383_c0_120897 | aaaacuuuuaguagaauauuuggg | 233 | 24 |
| novel-miR21 | unconservative_comp113142_c0_32082 | auugacggauuggagaaaaucaau | 143 | 24 |
| novel-miR22 | unconservative_comp149863_c0_265735 | ccauuugagccacagaauuug | 30 | 21 |
| novel-miR23 | unconservative_comp138578_c0_198106 | cccuucaaagacucugacugg | 24 | 21 |
| novel-miR24 | unconservative_comp48358_c0_139912 | uggaauguugggucuuugagggug | 21 | 24 |
| novel-miR25 | unconservative_comp155832_c0_186239 | ucugugugcuauuaugugaaa | 22 | 21 |
| novel-miR27 | unconservative_comp61995_c0_87861 | ugccaugccgcggccuuggccggc | 27 | 24 |
| novel-miR28 | unconservative_comp138428_c0_22691 | auauuaaugacauucaugugg | 12 | 21 |
| novel-miR29 | unconservative_comp157526_c0_56906 | agguacuuucagagggagca | 15 | 20 |
| novel-miR30 | unconservative_comp97259_c0_41591 | uugaguaaagauuuguagagg | 9 | 21 |
| novel-miR31 | unconservative_comp161142_c1_309708 | agcgugacugggauuacaugaucc | 9 | 24 |
| novel-miR32 | unconservative_comp136336_c0_51451 | aucugagaucugucguccucucuc | 9 | 24 |
| novel-miR33 | unconservative_comp152359_c0_9457 | ugagcucgagcguagauuagcagg | 21 | 24 |
| novel-miR34 | unconservative_comp139276_c0_308631 | ucuguguccgguuuuguguuc | 9 | 21 |
| novel-miR35 | unconservative_comp42393_c0_242624 | uuguugacagaagauagagagc | 20 | 22 |
| novel-miR36 | unconservative_comp116569_c0_188359 | aucuuuggcaaguagaauagcagg | 11 | 24 |
| novel-miR37 | unconservative_comp162111_c0_118490 | augugacuauuguagaauauugug | 7 | 24 |
| novel-miR38 | unconservative_comp162809_c3_350528 | cucgaaacugucaaaacgacg | 30 | 21 |
| novel-miR39 | unconservative_comp154102_c0_142296 | auaucaacguggugugucuguuac | 9 | 24 |
| novel-miR40 | unconservative_comp151190_c0_14349 | cauguaagagguuugguaauu | 4 | 21 |
| novel-miR41 | unconservative_comp162802_c2_305945 | aaaucuacgcucgagcggacuggu | 9 | 24 |
| novel-miR42 | unconservative_comp48705_c0_327902 | gguucuaaauaucggccgauacgu | 8 | 24 |
| novel-miR43 | unconservative_comp140469_c0_20771 | aauaguucgcuuugugcucacc | 5 | 22 |
| novel-miR44 | unconservative_comp161949_c0_87301 | ucagggggguaucuguaauuu | 9 | 21 |
| novel-miR45 | unconservative_comp116755_c0_39153 | ccacugccacaucacuagu | 2 | 19 |
| novel-miR17 | unconservative_comp161812_c1_32660 | cgguugcggguugacuuuuuc | 4 | 21 |
| novel-miR3 | unconservative_comp160443_c2_144034 | acgguuccucugacagcagucgug | 3 | 24 |
| novel-miR5 | unconservative_comp154814_c0_69223 | gcuucaggucacggugguagacgc | 2 | 24 |
| novel-miR26 | unconservative_comp146627_c0_290056 | ucaaaucacucacaaccauuggau | 3 | 24 |
