# Supplementary material for: Combined small RNA and degradome sequencing reveals complex microRNA regulation of catechin biosynthesis in tea (Camellia sinensis)
Source: PLoS One. 2017 Feb 22;12(2):e0171173. doi: 10.1371/journal.pone.0171173 (PMC5321428; doi:10.1371/journal.pone.0171173)
Supplement: S6 Table — (DOC) [file pone.0171173.s006.doc]

**S6 Table. Potential miRNAs regulating catechins biosynthetic pathway genes**

| **target** | **ID** | **miRNA** | **Expectation** | **UPE** | **Inhibition** | **Multiplicity** |
| --- | --- | --- | --- | --- | --- | --- |
| Chalcone isomerase (CHI) | DQ904329.1 | Csn-miR167a | 4.5 | 15.579 | Translation | 1 |
| Anthocyanidin synthase (ANS) | AY830416.1 | Novel-miR26 | 5 | 24.005 | Translation | 1 |
| p-coumaroyl-CoA ligase 1(4CL) | comp139381_c0 | Csn-miR5498 | 5 | 24.415 | Cleavage | 1 |
|  |  | Novel-miR8 | 4 | 21.768 | Cleavage | 1 |
| p-coumaroyl-CoA ligase 2(4CL) | comp141849_c0 | Csn-miR5023 | 5 | 13.747 | Translation | 1 |
|  |  | CNovel-miR40 | 5 | 13.802 | Translation | 1 |
| Cinnamate 4- hydroxylase (C4H) | comp154342_c0 | Csn-miR7777-5p.1 | 4.5 | 15.496 | Translation | 1 |
|  |  | Csn-miR5251 | 5 | 18.695 | Cleavage | 1 |
| CHI | comp108537_c0 | Csn-miR167a | 4.5 | 15.488 | Translation | 1 |
| Anthocyanidin reductase 1 (ANR) | comp146498_c0 | Csn-miR7717c-3p | 4.5 | 20.219 | Cleavage | 1 |
| Anthocyanidin reductase 2 (ANR) | GU992400 | Csn-miR2593e | 4 | 14.058 | Cleavage | 1 |
| Leucoanthocyanidin reductase (LAR) | comp149372_c0 | Csn-miR426c | 5 | 11.328 | Cleavage | 1 |
|  |  | Csn-miR1520e | 5 | 15.392 | Cleavage | 1 |
|  |  | Csn-miR426b | 5 | 6.976 | Translation | 1 |
| Dihydroflavonol 4-reductase (DFR) | comp136300_c0 | Csn-miR3444b | 4.5 | 15.208 | Cleavage | 1 |
|  |  | Csn-miR4380a | 4.5 | 4.119 | Cleavage | 1 |
|  |  | Novel-miR28 | 4 | 17.008 | Translation | 1 |
