# Supplementary material for: Combined small RNA and degradome sequencing reveals complex microRNA regulation of catechin biosynthesis in tea (Camellia sinensis)
Source: PLoS One. 2017 Feb 22;12(2):e0171173. doi: 10.1371/journal.pone.0171173 (PMC5321428; doi:10.1371/journal.pone.0171173)
Supplement: S1 Appendix — (DOC) [file pone.0171173.s007.doc]

Abbreviations list

**5-RLM-RACE:** RNA ligase-mediated rapid-amplification of 5’cDNA ends;

**ANS:** Anthocyanidin synthase;

**ANR:** Anthocyanidin reductase;

**ARF:** Auxin response factor; Bhlh: Basic helix-loop-helix;

**CDS:** Coding sequence;

**CHS:** Chalcone Synthase;

**CHI:** [Chalcone isomerase](javascript:showjdsw('showjd_0','j_0'));

**C4H:** Cinnamate 4- hydroxylase;

**DFR:** Dihydroflavonol 4-reductase;

**ECG:** Epicatechins gallate;

**EGCG:** Epigallocatechins gallate;

**EC:** Epicatechins;

**EGC:** Epigallocatechins;

**ERF:** Ethylene-responsive transcription factor;

**EST:** Expressed sequence tag;

**F3’5’H:** Flaconoid3’, 5’-hydroxylase;

**GRF:** Growth-regulating factor;

**GO:** Gene ontology;

**HPLC:** High Performance Liquid Chromatography;

**KEGG:** Kyoto encyclopedia of genes and genomes;

**UTR:** Untranslated region;

**QRT-PCR:** Quantitative real-time PCR.
